# Clear-cell carcinoma of the soft palate: a case with atypical presentation and long-term follow-up: a case report

**DOI:** 10.1186/s13256-025-05115-3

**Published:** 2025-02-28

**Authors:** Sardar Noman Qayyum, Sayyed Muddasir Shah, Irfan Ullah, Maleeka Khan, Gulmeena Aziz Khan, Safi Ullah, Samim Noori, Mudassir Hussain

**Affiliations:** 1 Bacha Khan Medical College, Mardan, Pakistan; 2 Faculty of Medicine, Nangarhar University, Nangarhar, Afghanistan; 3 Shaukat Khanum Memorial Cancer Hospital, Lahore, Pakistan

**Keywords:** Clear-cell carcinoma, Soft palate, Epithelial tumors, Head and neck cancers

## Abstract

**Background:**

Clear-cell carcinoma, a rare malignancy of the minor salivary gland of the soft palate, is diagnostically challenging neoplasm due to its rarity and overlapping features with other neoplasms. We report a case of atypical presentation, diagnostic challenges, and long-term follow-up post-surgical resection of the tumor, which adds valuable insights to literature on this rare malignancy.

**Case presentation:**

A 34-years-old Pakistani female came to the hospital with a 2 × 2 cm ulcerative lesion of soft palate. The lesion had no active bleeding and any associated discharge. However, during history taking, she reported pain and occasional bleeding from the lesion.

**Diagnosis and therapeutic interventions:**

Excisional biopsy was performed, and the specimen was sent for histopathological examination and immunohistochemistry, which confirmed the diagnosis of clear-cell carcinoma. Later on, radiological evaluation confirmed the diagnosis of hyalinizing variant of clear-cell carcinoma. Long-term follow-up revealed no recurrence and postoperative complications.

**Conclusion:**

This case report highlights the importance of thorough diagnostic evaluation and long-term follow-up in management of a rare oral malignancy. Histopathological examination and immunohistochemistry are crucial in differentiating clear-cell carcinoma from other malignancies with overlapping features. Surgical excision remains the primary treatment modality, with a favorable prognosis if diagnosed and managed adequately.

## Introduction

Clear-cell carcinoma (CCC) is an extremely rare malignancy arising mostly from minor salivary glands in the oral cavity. It is a low-grade tumor composed of glycogen-rich monomorphic tumor cells with cell to eosinophilic cytoplasm, arranged in cords, nests, and sheets in a fibrous or hyalinized stroma [1]. It accounts for less than 1% of all salivary gland tumors with more female predominance [2]. CCC requires meticulous diagnostic evaluation, as it is often misinterpreted as mucoepidermoid carcinoma, squamous-cell carcinoma, clear-cell odontogenic carcinoma, epithelial myoepithelial carcinoma, and metastatic clear-cell renal carcinoma due to overlapping histological features [3]. It may be asymptomatic and grows to a large size without causing discomfort [4]. It may be found on routine dental examination. Mucosal ulceration, pain, and bleeding are more uncommon presentation. More than 80% of the intraoral CCCs occurs in minor salivary glands, while a minority may also occur in major salivary glands and the nasopharynx. The diagnosis can be made through histopathological examination and immunohistochemical staining [3]. In addition to it, radiological examinations may also be needed to excluded potential differentials. Complete surgical excision with clear margins is the primary treatment modality, radiotherapy is reserved for cases exhibiting local metastasis. Given the rarity of the malignancy and the limited number of reported cases with short-term follow-ups, its biological behavior is not fully understood. In this case report, we discuss an usual presentation of hyalinizing clear-cell carcinoma in a young female, presenting with palatal growth with pain and sporadic bleeding. The important learning points discussed are diagnostic workup and patient outcomes from long-term follow-up.

## Case presentation

### Patient demographics and clinical history

A 34-year-old Pakistani female presented to the otorhinolaryngology department of Ahmad Medical Complex, Mardan, with primary complaints of palatal growth associated with sporadic pain and bleeding. The patient had no significant past medical, psychosocial, and family history, and no such intervention had been performed before this malignancy.

### Clinical examination

On examination, a 2 × 2 cm ulcerative lesion was observed on the soft palate, covered centrally by a pseudo-membrane. The lesion was non-indurated, with no active bleeding or discharge at the time of examination. No other significant findings were seen on the head and neck examination. There were no palpable lymph nodes or facial asymmetry. Table 1 summarizes the whole timeline of the case.

**Table 1:** Timeline of the case

Initial presentation	A 34-year-old female presented to the otorhinolaryngology department with complaints of palatal growth, sporadic pain, and bleeding.
Day 1: clinical examination	Examination revealed a 2 × 2 cm ulcerative lesion on the soft palate, covered by a pseudomembrane. No palpable lymph nodes or other abnormalities.
Day 2: initial management	Patient was prescribed oral antibiotics (ciprofloxacin) and tranexamic acid to manage sporadic bleeding.
Within 1 week: baseline investigations	Baseline investigations were advised and reviewed before proceeding with further interventions.
Excisional biopsy (scheduled)	Excisional biopsy of the lesion was performed under general anesthesia. The resected specimen was sent for histopathological evaluation.
Histopathological evaluation	Specimen revealed large polygonal clear cells with vesicular nuclei, and the immunohistochemistry confirmed clear-cell carcinoma (CCC)​.
Postsurgical follow-up (1 week)	Follow-up examination postsurgery showed no signs of complications, with the healing process being normal.
Postsurgical follow-up (1 month)	No signs of recurrence were observed during a routine follow-up visit.
Long-term follow-up (1 year and 8 months)	Regular follow-ups continued, with no evidence of local recurrence or metastasis during this period.

### Initial management

Baseline investigations were advised prior to any further intervention. The patient was started on oral antibiotics (ciprofloxacin) and tranexamic acid to manage the sporadic bleeding. The patient was informed about the excisional biopsy for the possibly neoplastic lesion. After taking the informed consent, baseline investigations were reviewed, and excisional biopsy was scheduled under general anesthesia.

### Histopathological evaluation

Once surgically resected, the specimen was sent to Shaukat Khanum Memorial Cancer Hospital and Research Centre, Lahore, for histopathological evaluation by Dr. Mudassir Hussain.

1. *Gross examination* The specimen was a disoriented tissue measuring 25 mm × 20 mm × 15 mm. Serial slicing revealed a soft to firm, tan-white, homogenous cut surface extended to the margins.
2. *Microscopic examination* The microscopic examination revealed lobules of large polygonal clear cells with distinct cell borders, vesicular nuclei, and inconspicuous nucleoli. Sclerotic areas were seen separating the lobules.

### Immunohistochemistry

The immunohistochemistry staining was performed, which yielded the following results:

- *CK7* positive
- *p63* positive
- *PAS, PASD, mucin, S100* negative

### Diagnosis

The histological and immunohistochemical features, combined with the clinical presentation, were consistent with a diagnosis of clear-cell carcinoma (CCC). The differential diagnosis included: (1) hyalinizing clear-cell carcinoma of the salivary gland (HCCC) and (2) clear-cell odontogenic carcinoma. Further radiological examination revealed the presence of salivary origin, confirming the diagnosis of HCCC. The patient faced no testing, financial, or cultural challenges during the diagnostic procedure,

### Management and follow-up

**Fig. 1:**
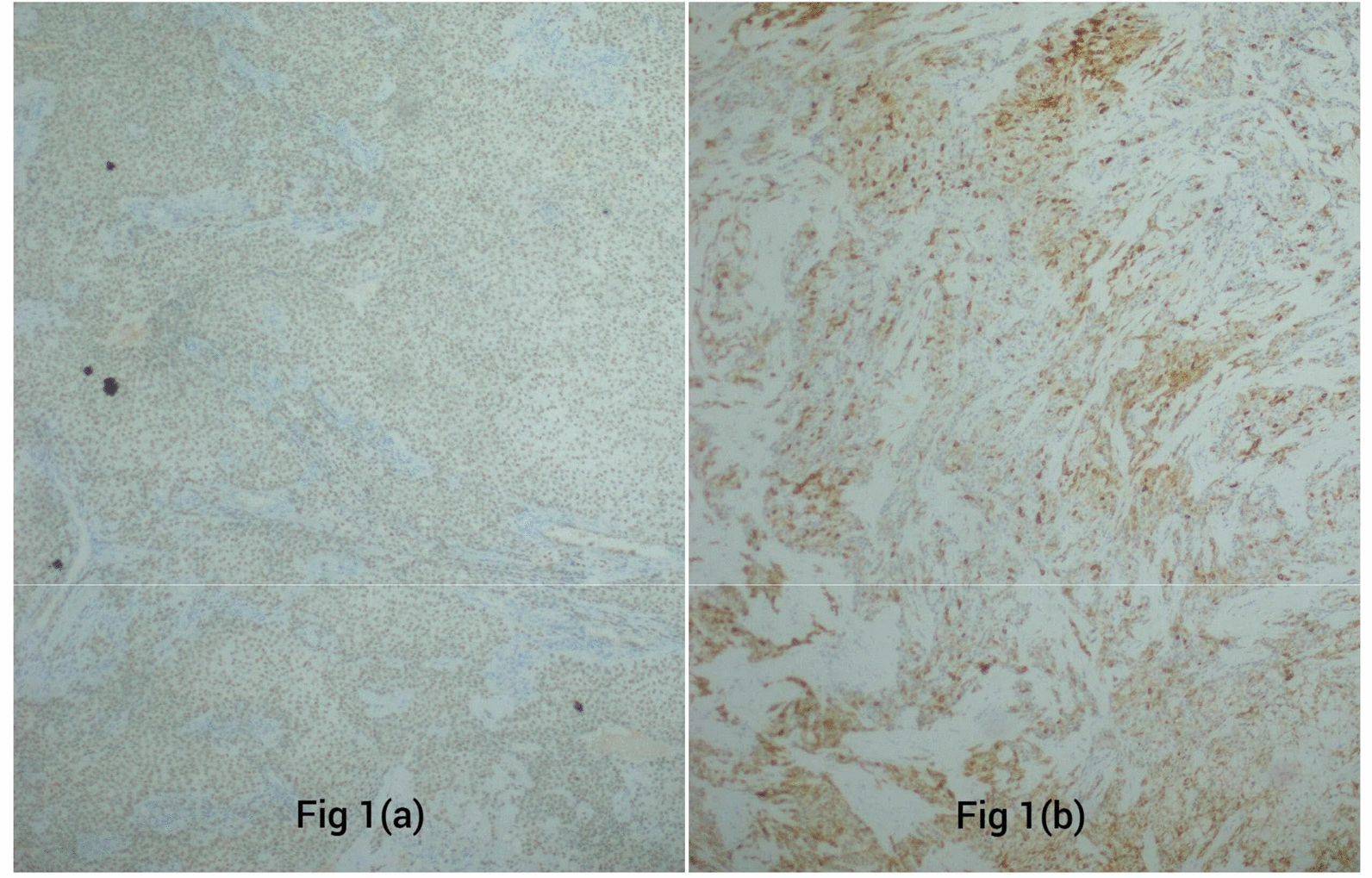
Immunohistochemistry of the specimen (**a**). Tumor cells positive for p63 (**b**). Tumor cells strongly positive to the CK7 stain

**Fig. 2:**
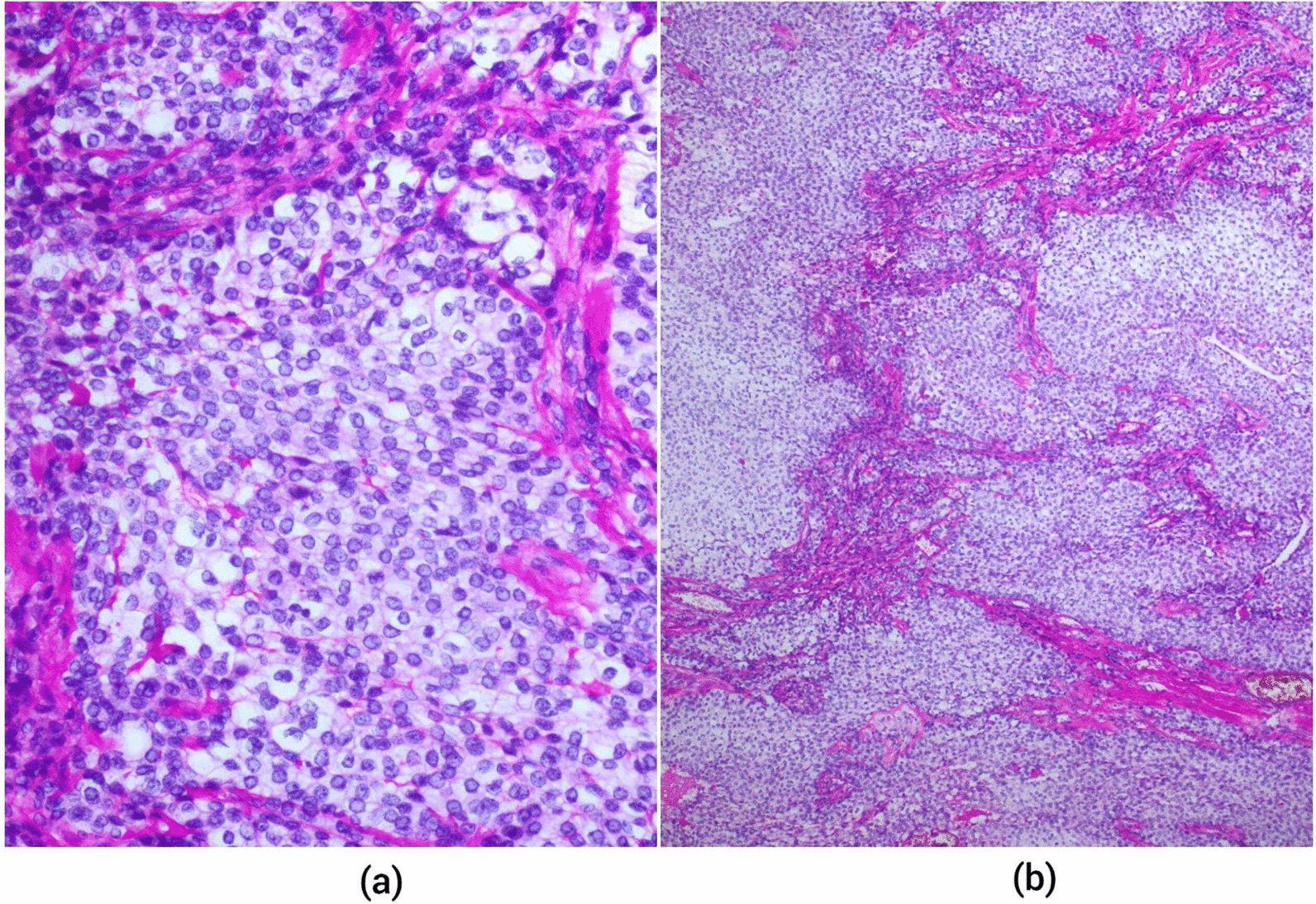
Histology of the specimen (**a**). High-power histological image of the specimen (**b**). Low-power histological image of the specimen

**Fig. 3:**
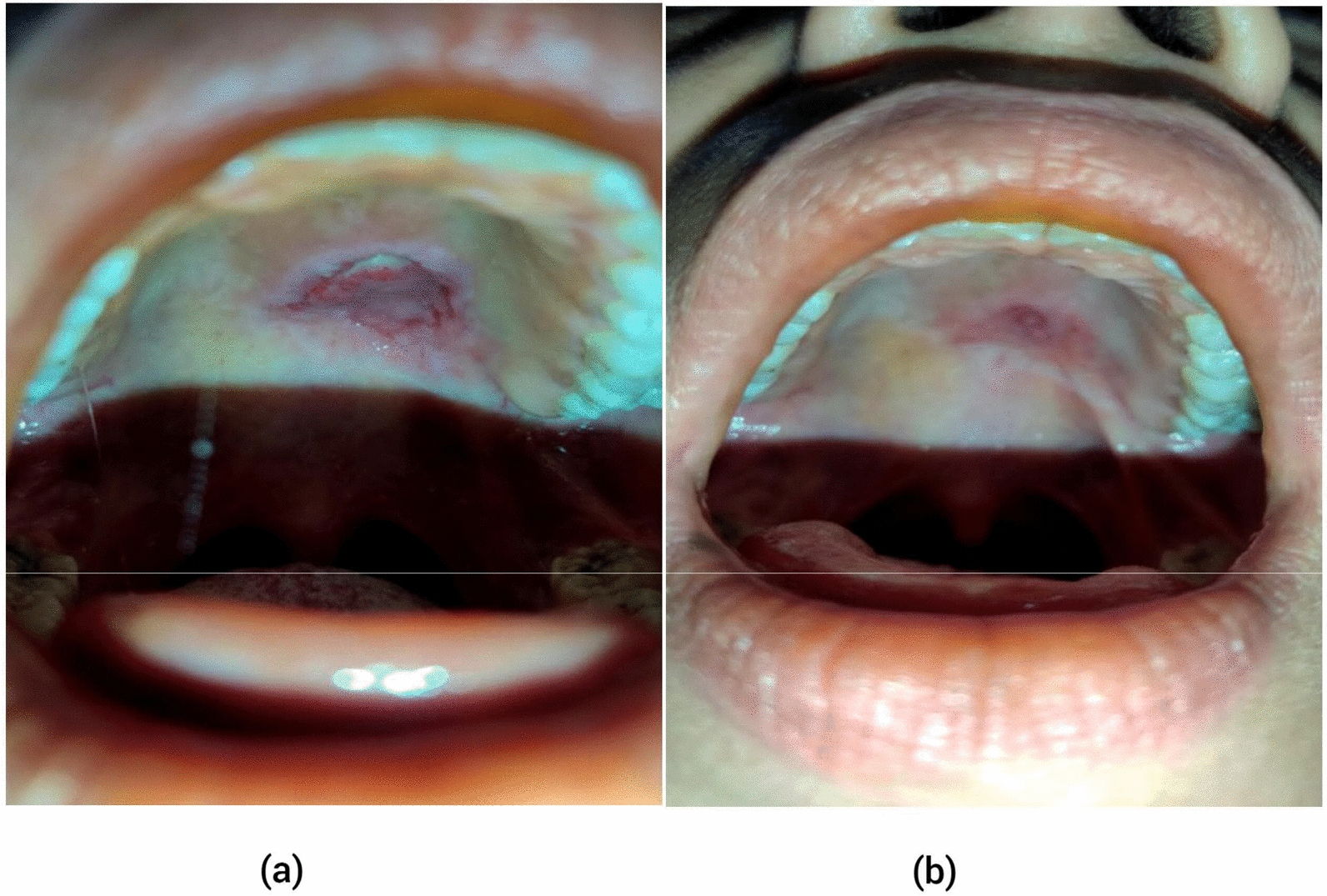
Intraoral view of the soft palate post-surgical resection (**a**) after 1 week and(**b**) after 1 month demonstrating progressed healing

The lesion was surgically resected, and the patient has been under regular follow-up for the preceding 1 year and 8 months. No evidence of local recurrence or metastasis or any adverse event has been observed during this period. Figure 1 demonstrates the immunohistochemistry evaluation of the tumor cells, Fig. 2 presents the histology of the specimen, and Fig. 3 demonstrates the healing phases 1 week postsurgery (3a) and 1 month postsurgery (3b).

## Discussion

Clear-cell carcinoma (CCC) is an exceptionally rare salivary gland malignancy. First reported in 1994 by Milchgrub, it is found predominantly in the intraoral minor salivary glands and elderly females [5]. The term “clear-cell carcinoma not otherwise specified” (CCC-NOS) was officially introduced in 2005 in the fourth edition of the World Health Organization classification of salivary neoplasms. Later, it was further revised in 2017 [5]. It is a low-grade tumor, with low mitotic activity and good prognosis. A literature review reported 254 cases between 1983 and 2020, with the palate as the most common site followed by the tongue. CCC accounts for 0.2–1% of all salivary gland tumors with slight female predominance. It mainly affects minor salivary glands (> 80% of the cases) [5]. The majority of the CCCs present as painless, slow-growing masses, with patients typically being asymptomatic, and may be diagnosed incidentally on regular dental checkup. However, in this case, the patient reported atypical symptoms, including pain and sporadic bleeding. Other less common symptoms are dysphagia, foreign body sensations, and tenderness [3]. The etiology is not known, with keratin and p63 positivity indicating squamous-cell differentiation, and it could be part of the spectrum of squamous-cell carcinomas [6]. However, the presence of occasional glandular differentiation (50% of the cases) and mucin production is suggestive of a more complex histogenesis, and the tumor may arise from pluripotent cells [7]. Microscopically, the presence of monomorphic tumor cells with clear or eosinophilic cytoplasm arranged in anastomosing nests, trabeculae, or cords in the hyalinized or myxoid stroma is the hallmark of clear-cell carcinoma. The tumor may have pseudoepitheliomatous hyperplasia mimicking squamous-cell carcinoma and can be found in association with surface epithelium in a pagetoid pattern [8]. The tumor can also invade the bones and nerves in advanced stage and can be difficult to treat with higher rates of recurrence [8].

The diagnosis of CCC variants can be challenging due to their histopathological overlap with other neoplasms such as mucoepidermoid carcinoma, acinic-cell carcinoma, clear-cell odontogenic carcinoma, and metastatic renal-cell carcinoma [3]. Immunohistochemistry plays a crucial role in distinguishing CCCs from other clear-cell malignancies. In this case, the positive immunohistochemistry staining for CK7 and p63 and the negative staining for PAS, PASD, mucin, and S100 and mammaglobin helped rule out other neoplasms such as mucoepidermoid carcinoma, and epithelial myoepithelial carcinoma, metastatic renal clear-cell carcinoma, clear-cell myoepithelial carcinoma, and other mammary-type tumors. However, it was suspected that the patient might have two variants of CCC: hyalinizing clear-cell carcinoma (salivary gland origin) and clear-cell odontogenic carcinoma (bone origin). Radiological correlations were examined to rule out the bone origin. The clinical presentation for both almost similar except for the bone involvement and loosening of the teeth observed in odontogenic variant of CCC. Therefore, given the rarity of the CCCs, careful histopathological, immunohistochemistry, and radiological evaluations are necessary to avoid misdiagnosis.

Complete surgical excision with clear margins is the primary treatment. In literature, safe margins of 0.5–2 cm have been recommended, depending on the extent of the tumor and its proximity to critical anatomical structures [9]. In our case, the patient underwent complete local excision with a clear margin, as determined by the intraoperative assessment and postoperative histopathological examination. The resection was curative, and no additional therapy such as radiotherapy or chemotherapy was needed, as the tumor had not invaded critical structures. Postoperative complications can include oral–nasal fistulas, which can lead to nasal regurgitation or changes in taste and smell. In cases where a palatal defect is created postresection, reconstruction using a buccal fat pad is often necessary to restore normal function. Fortunately, in this case, the surgical procedure was uneventful. As per the literature, CCCs have local recurrence rate of 18.8% [10] and metastatic potential of 21% [2]; therefore, close follow-up is recommended for a longer period. In this case, the patient has been followed up for 1 year and 8 months postsurgery with no signs of recurrence and postsurgical complications. This supports the notion that CCC is a low-grade carcinoma with limited metastatic potential.

## Conclusion

Clear-cell carcinoma of the soft palate is an exceedingly rare, low-grade malignancy with a good prognosis and low recurrence rates if diagnosed and surgical excised appropriately. CCC can occasionally manifest with atypical symptoms, as seen in this case, where a young female presented with palatal growth, pain, and sporadic bleeding. Histopathological examinations and immunohistochemical staining remain the important diagnostic elements for the diagnosis of different CCC variants. The positive staining for CK7 and p63 and negative staining for PAS, PASD, mucin, and S-100, and radiological evaluation helped rule out potential differentials and confirmed the diagnosis of hyalinizing variant of CCC.

Surgical excision with a clear margin is the treatment of choice. Through this case, we want to emphasize the necessity for careful diagnostic workup and long-term follow-up for monitoring recurrence.

## Data Availability

The data are available through the corresponding author, and can be provided on request.
